# Climate change impact on blood haemogram in the horse: a three-year preliminary study

**DOI:** 10.3389/fvets.2024.1482268

**Published:** 2024-12-16

**Authors:** Ömer Deniz, Francesca Aragona, Barbara A. Murphy, Kenan Çağrı Tümer, Serkan Bozacı, Francesco Fazio

**Affiliations:** ^1^Faculty of Veterinary Medicine, Department of Clinical Science and Internal Medicine, Kastamonu University, Kastamonu, Türkiye; ^2^Department of Veterinary Sciences, University of Messina, Messina, Italy; ^3^School of Agriculture and Food Science, University College Dublin, Dublin, Ireland

**Keywords:** horses, climate changes, haemogram, seasonal variations, hematology

## Abstract

**Introduction:**

The global climatic changes pose a substantial threat to the well-being and productivity of both humans and animals.

**Methods:**

This study examined the impact of climate changes during different seasons over a 3-year monitoring period (2021–2023) on various blood parameters including, white blood cells (WBC), neutrophils, basophils, eosinophils, lymphocytes, and monocytes, hematocrit (HCT), hemoglobin (HGB), red blood cells (RBC), platelets (PLT), mean corpuscular hemoglobin concentration (MCHC), mean corpuscular volume (MCV), and mean corpuscular hemoglobin (MCH). The study focused on 25 Thoroughbred mares located in Kastamonu-Türkiye. Thermal and hygrometric parameters, including ambient temperature, relative humidity, and ventilation, were collected. Subsequently, Temperature-Humidity index (THI) was computed. Blood samples were collected on the first day of every month from January 2021 to December 2023 and used for a complete blood count analysis. Between 2021 and 2023, changes in environmental indicators were correlated to changes in hematological parameters.

**Results:**

Two-way for repeated measures ANOVA revealed a significant seasonal fluctuation (<0.0001) in ambient temperature, relative humidity, and THI. There was a reduction in RBC (<0.01), and MCH (<0.01) every year, HGB (<0.0001) in summer 2021, 2022 and in summer and autumn 2023. HCT (<0.0001), MCV (<0.01), showed decreasing values in autumn 2022 and 2023. MCHC values showed increasing values in July and August 2021, 2022 and in June 2023. WBC levels increased throughout the spring periods of 2021 and 2022. In April 2021, there were elevated levels of lymphocytes and monocytes (<0.0001) respectively.

**Discussion:**

These findings could be helpful to promote the monitoring of physiological status both for the assessment of welfare status and for diagnostic purposes for the evaluation of possible disease outbreaks due to climate change in veterinary medicine.

## 1 Introduction

Currently, global temperature trends and weather conditions are showing long-term deviations from historical seasonal average values and temperatures are predicted to continue to rise steeply in the coming decades (1). Seasonal variability in temperature, humidity, wind and rainfall, has been today classified as a potential risk to human welfare, animal growth and production (2). In fact, climate change is widely recognized as one of the most significant challenges facing the planet, for both humans and animals (3). Climate change and global warming is responsible for a higher frequency of extreme weather events and changing conditions, such as intense rainfall, frost, prolonged heat cycles, and prolonged drought (4–6). These effects negatively influence the viability, sustainability, productivity and reproduction of animal species, as well as compromising sports performance in horses (7). Direct effects of climate change resulting from increased greenhouse gasses such as carbon dioxide (CO2) concentrations influence mammalian thermoregulation, metabolism, immune system function and production (8–10). Indirect effects negatively influence feed production, water availability and parasite/pathogen populations (11, 12).

It was previously shown that severe weather events negatively affect the training and transport of athletic horses (13–17). Further literature suggested that climate change is increasing the risk of some diseases in domestic animals through increasing abundance of wildlife vectors and reservoirs, the survival of pathogens in the environment and husbandry practices (18). Horses are increasingly affected by respiratory diseases caused by smoke and dust from wildfires, skin diseases, hoof damage, parasites and emerging diseases, as a result of weather variability. In addition, drier and warmer conditions have been associated with an increase in bacterial infections associated with fecal contamination of dry soils (19).

Many implications of climate change may be observed and preliminarily recognized through changes in the hematological profile of animals. The influence of seasonal variations on hematological parameters has been largely studied in dairy cows (20–22), sheep (23), goats (24) and horses (9, 25, 26). Blood is a very dynamic tissue and its primary responsibility is the maintenance of body homeostasis in various conditions (25, 27). Hematological analysis is essential for monitoring general health conditions of individuals, to confirm clinical diagnoses and to monitor disease evolution and recovery during treatments (28–30).

In athletic horses, it serves as an optimal indicator to assess performance and to evaluate an animal’s physiological adaptability to exercise and to adverse environmental conditions (26–32). In domestic animals, hematological parameters depend on several factors including age, sex, breeds, diet, exercise, reproductive status, housing system and microclimatic conditions (25, 33–38). The knowledge of hematological physiology is an important tool that can be used as an effective and sensitive index to monitor physiological and pathological conditions in horses (39).

Hematological parameters are subject to periodic variations associated with biological rhythms in a number of species, including humans and horses (25, 40–43). Oscillations of biological functions, sustained with a period of about 1 day, are called circadian rhythms. Other endogenously generated biological rhythms include circatrigintan rhythms (with a period of about a month) and circannual rhythms (with a period of about 1 year) (13, 44).

All biological rhythms reflect the ability of endogenous adaptative mechanisms of animals to react in advance to environmental changes associated with daily and seasonal environmental changes (9, 39). Seasonality has an impact on several biological and physiological mechanisms in domestic animals based on periodic changes of photoperiod, nutritional availability, relative humidity and temperature that regulate changes in reproductive activity, pelage, lactation and metabolism (43, 45–48). The evaluation and analysis of seasonal changes in physiological parameters helps develop a better understanding of the effects of thermal changes on physiology and associated adaptation/acclimatization in mammals (49).

The objective of this study was to evaluate the possible variation in the hematological profile of horses in relation to seasonal climatic changes over a three-year period. This could potentially offer a picture of how changing environmental exposures associated with climate change and season impact important physiological parameters that can have an influence on welfare, diagnosis and performance.

## 2 Materials and methods

This study was approved by Kastamonu University Animal Experiments Local Ethics Committee and an approval certificate (Decision no: 2024/24) was obtained.

The data obtained during the monthly health status check of a regional breeding farm in Kastamonu city, Turkey, were used to conduct this retrospective study. Twenty-five healthy, non-pregnant retired Thoroughbred mares aged between 9 and 20 years old and with a mean body weight of 558 ± 20 kg were randomly chosen to be enrolled in the study. The mares were housed in individual stables, each measuring between 6 and 12 square meters. They were turned out to adjacent paddocks for a number of hours depending on seasons and weather conditions and were managed by experienced stable staff. Mares were located at Golkoy Breeding Farm l in Kastamonu-Turkey (Latitude: 41.371; Longitude: 33.7756; Altitude: 800.0 m). Prior to the start of the study, each horse underwent physical examination to establish health status (body temperature, heart rate, respiratory rate). During the experimental period the horses in the study had regular health checks from the same veterinarian, thus, none of the study horses were replaced or got sick. Routine deworming and vaccinations were delivered at weeks apart from the blood sampling, to avoid any confounding effects on the blood parameters caused by immune activation.

Mares were fed twice a day at 06:00 and 18:00 with good quality hay and concentrates: 44% oatmeal (2.64 kg), 24% crushed barley (1.44 kg), 12% maize (0.72 kg), 15% soybean meal (0.9 kg), 4% bran (0.24 kg), salt, concentrated pellet feed (6 kg), dry grass (5 kg), lucerne (4 kg) and one mineral block. Water intake was available *ad libitum*. Thermal and hygrometric parameters (ambient temperature- °C; relative humidity- %; and ventilation- m/s) were obtained through access to the Kastamonu Meteorology Directorate to evaluate microclimatic conditions.

The temperature-humidity index (THI) was calculated according to the formula reported in Thom (70):

THI = [0.8 × *T* + (RH/100) × (*T* – 14.4) + 46.4] where *T* is the average ambient temperature expressed in °C and RH is the relative humidity expressed in % (50, 51).

### 2.1 Blood sampling and analysis

Blood samples were collected via jugular venipuncture into 4 mL vacutainer tubes containing EDTA-3K before the morning feeding (05:30) every first day of each month from January 2021 to December 2023. All blood samples were refrigerated at 4°C and analyzed for complete blood count (CBC) within 2 h. White blood cells (WBC), neutrophils, basophils, eosinophils, lymphocytes, and monocytes, hematocrit (HCT), hemoglobin concentration (HGB), red blood cells (RBC), platelets (PLT), mean corpuscular hemoglobin concentration (MCHC), mean corpuscular volume (MCV), mean corpuscular hemoglobin (MCH), were measured and counted using an automated hematology analyzer [HASVET VH5R Antalya, Türkiye (Norma iVet-5 device manufactured by Norma Diagnostika Budapest, Hungary)].

### 2.2 Statistical analysis

Normal distribution of the data was tested using Kolmogorov–Smirnov tests. Differences in hematological and environmental parameters for the independent variables of month and year were investigated using two-way repeated measures analysis of variance (ANOVA) followed by *post hoc* multiple comparison tests where appropriate. Percentage changes between study years in environmental and hematological parameters were calculated using Microsoft Excel 2021. Monthly values were first grouped by season (Dec, Jan, Feb = winter; Mar, Apr, May = spring; Jun, Jul, Aug = summer; and Sep, Oct, Nov = autumn) and mean values calculated. Regression lines between the monthly values of each hematological parameter and, 95% confidence interval for monthly mean max ambient temperature, chosen as a representative value, and relative humidity for all years were determined and Pearson correlation coefficient (*r*) was evaluated. Data were analyzed using statistical software Graph Pad Prism v. 9.5.1 (Graphpad Software Ltd., United States). Data were reported as means ± standard deviation (SD), a *p*-value less than 0.05 was considered statistically significant and *q*-value corresponding to the adjusted *p*-value (False Discovery Rate) was determined.

## 3 Results

**Figure 1:**
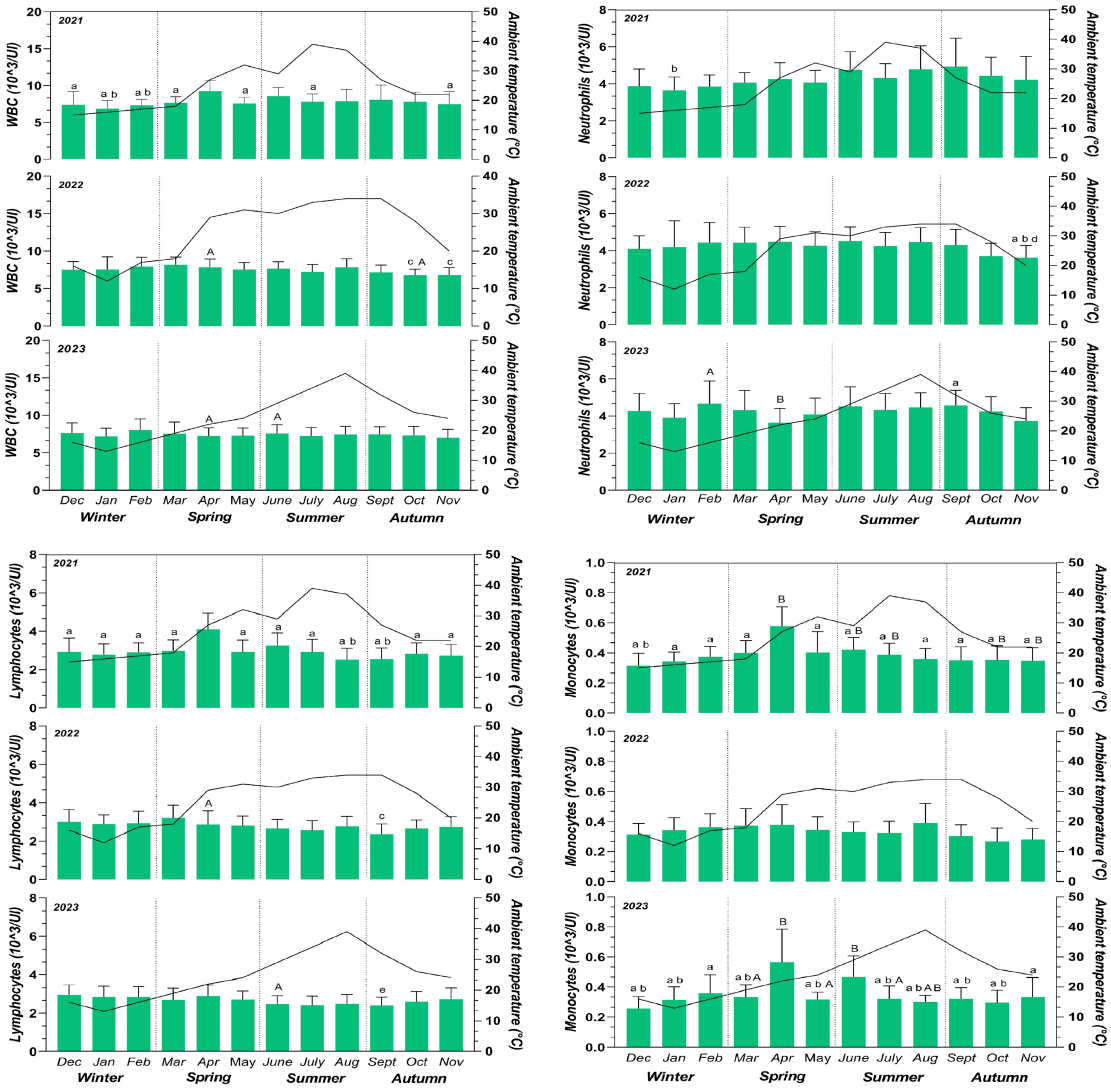
Mean values ± standard deviation (±SD) of WBC, neutrophils, lymphocytes and monocytes obtained from a monthly monitoring of 25 horses during a 3-year (2021–2023) period considering the four seasons with monthly fluctuations of the max ambient temperature during the monitoring period. Significances among months (*p* < 0.05): ^a^ vs. April; ^b^ vs. June, ^c^ vs. March, ^d^ vs. August and ^e^ vs. December significances among years (*p* < 0.05): ^A^ vs. 2021; ^B^ vs. 2022.

**Figure 2:**
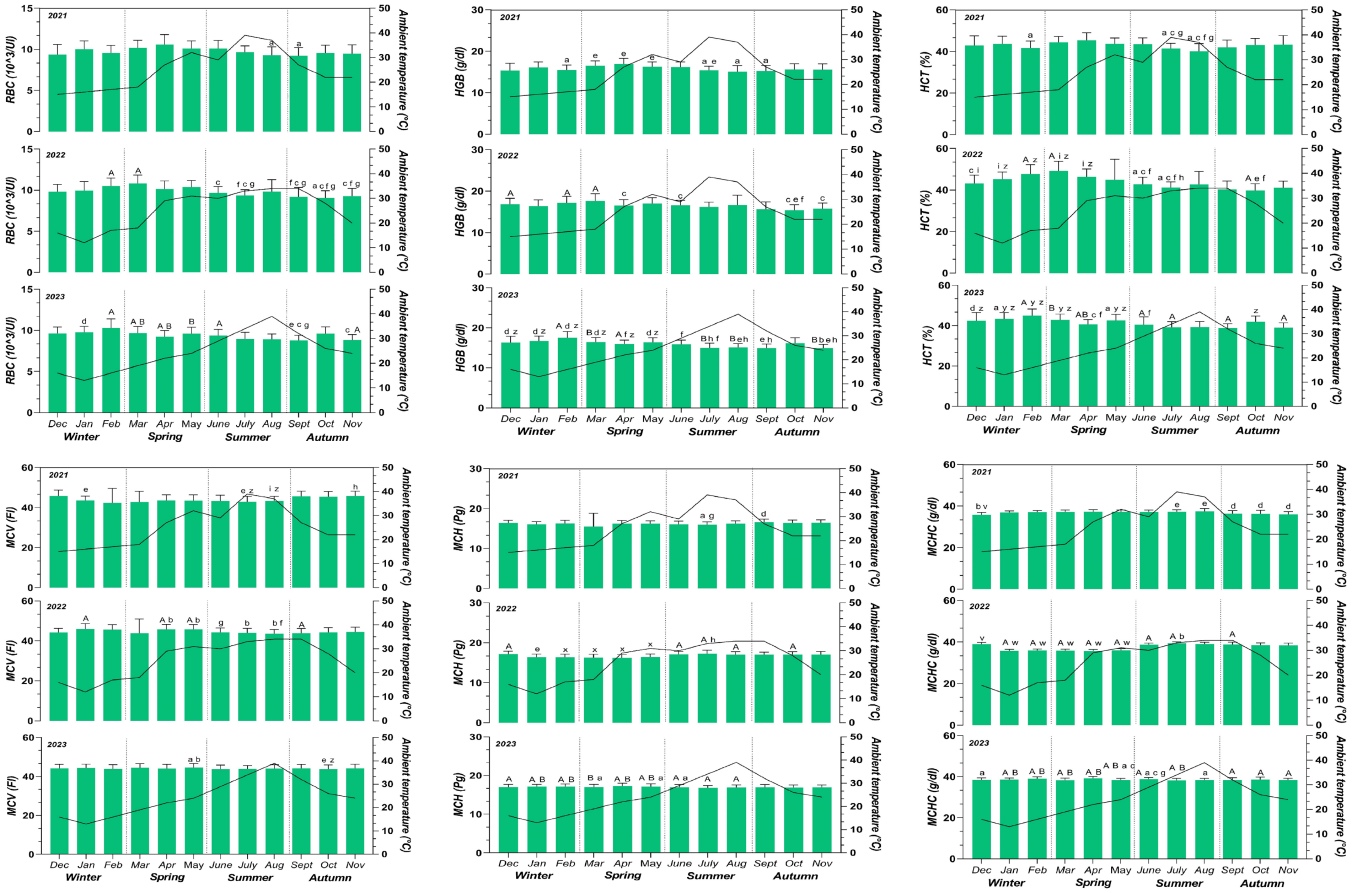
Mean values ± standard deviation (±SD) of RBC, HGB, HCT, MCV, MCH, MCHC obtained from a monthly monitoring of 25 horses during a 3-year (2021–2023) period considering the four seasons with monthly fluctuations of the max ambient temperature during the monitoring period. Significances among months (*p* < 0.05): ^a^ vs. April; ^b^ vs. June, ^c^ vs. March, ^d^ vs. August and ^e^ vs. December, ^f^ vs. February, ^g^ vs. May, ^h^ vs. January, ^I^ vs. October, ^z^ vs. September and November, ^y^ vs. July and August, ^x^ vs. June and July, ^v^ vs. January, February, March, April, May, ^w^ vs. June, July, August, September, October, and November significances among years (*p* < 0.05): ^A^ vs. 2021; ^B^ vs. 2022.

**Figure 3:**
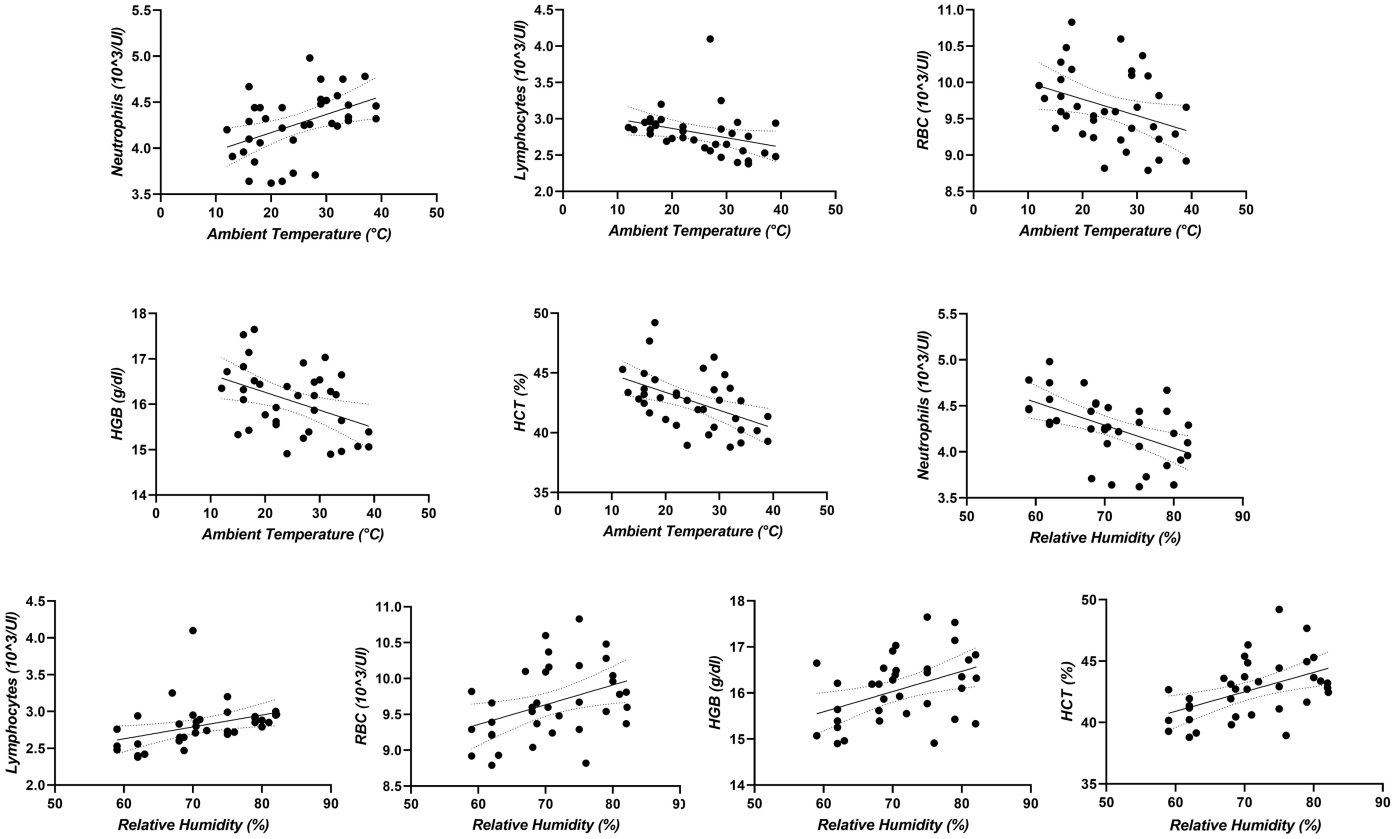
Regression lines and Pearson correlation coefficient (*r*) between the monthly values of hematological parameters and 95% confidence interval for monthly mean max ambient temperature, chosen as a representative value, and relative humidity for all years.

Data were not normally distributed (*p* < 0.05). The values for all hematological parameters were within the reference ranges of horses for the entire monitoring period (52). The two-way ANOVA was performed to analyze the effect of month and year on hematological and environmental parameters. A significant effect of month was observed for ambient temperature (*p* < 0.0001; *q*=), relative humidity and THI showed a significant effect of month (*p* < 0.0001). Multiple comparison of environmental parameters was reported in Table 1. No significant variation of month and year was observed for ventilation. The application of two-way ANOVA on the hematological parameters revealed a significant effect of month on WBC (<0.01), Neutrophils (Neutr) (<0.0001), Lymphocytes (Lymph) (<0.0001), Monocytes (Mon) (<0.0001), RBC (<0.01), HGB (<0.0001), HCT (<0.0001), MCV (<0.01), MCH (<0.01) and MCHC (<0.0001) and a significant effect of year on WBC (<0.01), Neutr (<0.01), Lymph (<0.0001), Mon (<0.0001), RBC (<0.01), HGB (<0.01), HCT (<0.0001), MCV (<0.01), MCH (<0.0001) and MCHC (<0.0001) as shown in Figures 1, 2. The percentage change of hematological parameters observed during the 3 years is shown in Table 2. A positive correlation was observed between monthly mean max ambient temperature and Neutr (*p* < 0.01: *r* = 0.45), and between relative humidity and Lymph (*p* < 0.01; *r* = 0.38), RBC (*p* < 0.01; *r* = 0.39), HGB (*p* < 0.01; *r* = 0.43) and HCT (*p* < 0.001; *r* = 0.48). A negative correlation was observed between monthly mean max ambient temperature and Lymph (*p* < 0.01; −0.33), RBC (*p* < 0.01; *r* = −0.35), HGB (*p* < 0.01; *r* = −0.37), HCT (*p* < 0.001; *r* = −0.48) and between relative humidity and Neutr (*p* < 0.001; *r* = −0.53) (Figure 3).

**Table 1:** Three year data for maximum, average and minimum values of ambient temperature and ventilation, relative humidity percentage and THI obtained from 2021 to 2023 across the four seasons, expressed in their conventional units.

Experimental conditions	Winter			Spring			Summer			Autumn		
	December	January	February	March	April	May	June	July	August	September	October	November
2021												
Ambient temperature (°C)												
*max*	15^p q r s^	16^p q r s^	17^p q r s^	18^p q^	27	32	29	39^a r^	37^a r^	27^r^	22	22
*avg*	1^p q r s^	1^p q r s^	2.25^p q r s^	7^p q r s^	13.75^p q^	14.75^q r^	19.5^r^	19.75^r^	14.5^r^	9.75^r^	6.5	2
*min*	-14	−15^p q r s^	−17^p q r s^	−8^p q r s^	−4^q^	−2^q^	4^r^	9^r^	9^r^	3	−2	−5
Relative humidity (%)	82^p q r s t^	80^p q r s^	79^p q r s^	75^p q r s^	70^q^	70^q r^	67	62^r^	59^r^	62^r^	68	72
Ventilation (m/s)												
*max*	13.1	12.4	10.7	11.1	10.9	13.1	11.7	11.3	10.6	10.9	11.3	11.5
*avg*	6.31	6.55	6.51	6.98	6.8	5.63	7.11	6.12	6.37	4.71	4.59	5.84
*min*	2.6	1.6	3.4	4	3.2	2.7	4.7	3.9	2.2	2.7	1.6	3.3
Temperature-humidity index (THI)	36.21^p q r s^	34.6^p q r s^	38.60^p q r s^	46.45^p q^	56.94^p q^	58.55^q r^	67.1^r^	65.51^r^	58.06^r^	51.32^r^	46.23	39.07
2022												
Ambient temperature (°C)												
*max*	16^p q r s^	12^p q r s^	17^p q r s^	18^p q^	29	31	30	33^a r^	34^a r^	34^r^	28	20
*avg*	4^p q r s^	−1.5^p q r s^	0.75^p q r s^	0^p q r s^	9.25^p q^	12.25^q r^	16.75^r^	18.75^r^	22.5^r^	16.5^r^	11	7.25
*min*	−4	−14^p q r s^	−11^p q r s^	−14^p q r s^	−2^q^	1^q^	8^r^	6^r^	12^r^	1	−2	−2
Relative humidity (%)	82^p q r s t^	80^p q r s^	79^p q r s^	75^p q r s^	70.51^q^	70.46^q r^	68.69^q r^	62^r^	59^r^	62^r^	68.1	75
Ventilation (m/s)												
*max*	7.6	12.3	8.5	11.7	13.8	10.5	10.2	9.1	8.8	8.5	9.5	10
*avg*	4.29	6.45	5.42	7.01	7.51	6.1	6.57	6.76	5.15	5.84	5.43	4.94
*min*	1.6	2.2	2.8	3.6	3.6	3.6	3.2	4.6	2.4	3.5	2.1	2.4
Temperature-humidity index (THI)	41.07^p q r s^	32.48^p q r s^	36.22^p q r s^	46.4^p q r s^	50.17^q^	54.68^q r^	62.15^r^	65.75^r^	69.09^r^	60.90^r^	52.88	47.14
2023												
Ambient temperature (°C)												
*max*	16^p q r s^	13^p q r s^	16^p q r s^	19^p q^	22	24	29	34^a r^	39^a r^	32^r^	26	24
*avg*	3.5^p q r s^	1.75^p q r s^	0.75^p q r s^	4.5^p q r s^	8.5^p q^	11.5^q r^	16.5^r^	19.75^r^	22.25^r^	18.75^r^	12	6.75
*min*	−7	−7	−10	−10	−1	−1	7	8	10	4	0	−7
Relative humidity (%)	82.13^p q r s t^	81^p q r s t^	79^p q r s t^	75^p q r s t^	71^q^	70.36^q r^	68.73^q r^	63^r^	59^r^	62^r^	68	76
Ventilation (m/s)												
*max*	11.3	9.9	13.4	11.5	11.4	10.9	7.6	11.7	9	8.9	10.7	22
*avg*	3.92	4.03	7.89	6.23	6.61	4.5	4.88	6.39	6.86	6.53	5.62	9.3
*min*	2.6	1.5	2	2.1	3.9	2	1.9	2.7	3.6	3.5	3.5	2.8
Temperature-humidity index (THI)	40.26^p q r s^	37.55^p q r s^	36.22^p q r s^	42.57^p q r s^	49.1^q^	53.57^q r^	61.05^r^	65.57^r^	68.83^r^	64.09^r^	54.37	45.99

Superscript letters denote significance of Bonferroni *post hoc* tests: ^p^ vs. May and June, ^q^ vs. July, August and September, ^r^ vs. October and November, ^s^ vs. April, ^t^ vs. February and March.

**Table 2:** Percentage variations in hematological parameters in horses between years for each season during the study period.

Experimental conditions	Winter	Spring	Summer	Autumn
2021–2022				
WBC (10^3/Ul)	8.79	−2.49	−3.27	−7
Neutrophils (10^3/Ul)	14.96	7.91	0.64	−1.16
Lymphocytes (10^3/Ul)	3.03	−10.06	−7.37	−0.73
Monocytes (10^3/Ul)	−9.66	−19.10	−9.87	−14.19
Eosinophils (10^3/Ul)	16.88	1.13	−14.85	9.64
Basophiles (10^3/Ul)	1.01	0.69	8	18.87
RBC (10^6/Ul)	1.86	2.19	−0.24	−1.49
HGB (g/dl)	3.56	3.30	6.17	1.44
HCT (%)	4.42	5.45	1.42	−5.06
MCV (Fl)	7.66	1.27	1.92	−3.04
MCH (Pg)	1.52	17.54	6.75	3.32
MCHC (g/dl)	−3.13	−3.69	4.81	6.74
PLT (10^3/Ul)	0.32	26.75	−0.03	−2.92
2022–2023				
WBC (10^3/Ul)	1.18	−5.66	−3.33	5.74
Neutrophils (10^3/Ul)	3.89	−7.44	−0.17	10.3
Lymphocytes (10^3/Ul)	1.14	−3.69	−6.58	−0.30
Monocytes (10^3/Ul)	−2.66	13.64	8.97	14.93
Eosinophils (10^3/Ul)	0.71	60.19	21.29	26.78
Basophiles (10^3/Ul)	18.40	24.53	−1.82	−2.26
RBC (10^6/Ul)	0.74	−8.96	−5.24	−0.90
HGB (g/dl)	5.26	−4.57	−6.73	−1.56
HCT (%)	−2.93	−9.18	−5.83	−1.05
MCV (Fl)	−3.44	3.03	0.11	−0.02
MCH (Pg)	4.70	1	−1.34	−0.43
MCHC (g/dl)	8.45	7.82	−1.44	−0.27
PLT (10^3/Ul)	5.35	−2.04	−2.25	0.05
2021–2023				
WBC (10^3/Ul)	7.72	−8.73	−7.49	−2.70
Neutrophils (10^3/Ul)	14.80	−1.24	−2.22	−3.91
Lymphocytes (10^3/Ul)	2.82	−16.34	−14.42	−1.88
Monocytes (10^3/Ul)	−8.04	−13.22	−5.71	−3.77
Eosinophils (10^3/Ul)	6.46	29.52	−0.58	31.06
Basophiles (10^3/Ul)	3.11	−9.33	−2.87	2.74
RBC (10^6/Ul)	2.21	−7.38	−5.68	−2.92
HGB (g/dl)	8.47	−1.73	−1.17	−0.44
HCT (%)	0.90	−5.32	−4.75	−6.36
MCV (Fl)	1.26	2.99	1.95	−3.11
MCH (Pg)	6.26	5.92	5.24	2.85
MCHC (g/dl)	−0.29	3.82	3.28	6.44
PLT (10^3/Ul)	1.37	18.95	−5.38	−4.41

Top panel percentage increases (positive values) and decreases (negative values) between 2021 and 2022. Middle panel: percentage increases (positive values) and decreases (negative values) between 2022 and 2023. Bottom panel: percentage increases (positive values) and decreases (negative values) between 2021 and 2023.

## 4 Discussion

Many areas are facing increasing pressure from the effects of climate change, such as rising temperatures, increased variability in rainfall, increased frequency of extreme events and increased carbon dioxide concentrations. These changes have been found to impact domestic animal performance, production and welfare (11). Referring to the impact of microclimate, ambient temperature is an important ecological physical and environmental stimulus (53). Extreme hot and cold ambient temperatures affect animals physiological adaptation responses (54). The THI was used to estimate the degree of thermal stress experienced by an animal (51). It can be categorized into mild (70–80), severe (80–85), and deadly stress zones (>85), reflecting environmental conditions (9). The present ambient temperature and THI recorded were within the thermoneutral zone for horses, thus external conditions were all considered comfortable for horses during the experimental period (9). The present results showed no significant variations between all environmental parameters over the 3 years monitored. According to Table 1, summer and autumn periods resulted in higher average ambient temperature compared to winter and spring. Previous studies observed that seasonal variations may affect hematological profile and welfare in horses (21, 22, 25, 32). During 2021 RBC values decreased in August and September. During 2022, significantly lower values were observed in June, July, September, October and November, and during 2023 in August, September and November compared to other months. HGB decreased in July, August and September 2021, during 2022 minimum HGB values were observed in April, June, October and November and in April, May, June, July, August, September and November 2023. HCT values showed a decrease in autumn months (September, October, and November) compared to winter (December, January, and February) and spring (March, April, and May) 2022 and 2023.

The present findings were in agreement with previous studies in horses (9, 39, 55, 56), sheep (57), cows (58, 59) and goats (45, 60). Values of MCV measured in 2021 and 2022 showed lower values during July and August and in October during 2023. MCH values showed a statistical decrease in July and August during 2021 and an increase in June and July during 2022. The MCV and MCH was observed to have lower values during summer as also observed in previous studies in horses (25) and donkeys (32), suggesting that this adaptation could be related to reduction in cellular oxygen requirements in order to reduce metabolic heat load (61). MCHC values showed increased values in July and August during 2021, 2022 and in June during 2023. These variations were previously considered as metabolic acclimation to the environmental conditions (26). Reduced temperatures during winter are related to increased sympathetic activity by increasing metabolic capacity and inducing mobilization of the spleen to release erythrocytes into the bloodstream by promoting erythropoiesis (62, 63). Enhanced sympathetic activity in winter could lead to increased spleen mobilization, with the release of blood into the bloodstream (63). Higher ambient temperature stimulated an hemodilution effect, in which water was diverted into the circulatory system for evaporative cooling in association with the thermoregulatory mechanism, which influences RBC, HGB and HCT concentration (31).

White blood cell values showed increasing values in April and March during 2021 and 2022. Similarly, Lymph and Mon show high values in April during 2021 and during 2023 for Mon confirming previous studies (49, 58). Stress associated with cold weather in winter may suppress the immune response (64). The overall increase in WBC during spring was related to an increase in lymphocytes, monocytes, neutrophils and eosinophils, likely caused by increased infestation and bites from pests and insects (65).

A significant effect of the year was observed for the studied parameters. Red blood cells showed an increase in February from 2021 to 2023. A decrease in RBC values during spring (March, April, and May) 2022–2023 of 8.96% and from spring 2021 to 2023 of 7.35% was observed according to Table 2. Hemoglobin showed an increase in February 2022 and 2023 compared to 2021 and an increase in December and March 2022. A decrease in HGB was observed in March, July, August, and November 2023 compared to 2022 and a decrease in April 2023 compared to 2021. Hematocrit values in 2022 increased in February and March and decreased in October compared to 2021. In 2023 compared to 2021, HCT values increased in February and decreased in April, July, September, and November and decreased in March and April compared to 2022. MCV showed an increase in February, April, May, and September 2022 compared to 2021. A 6.75% increasing of MCH was shown in October and December 2022 compared to 2021 and in summer (June, July, and August). A statistical increase in MCH values in winter (December, January, and February) by 6.26%, spring (March, April, and May) by 5.92%, and summer (June, July, and August) by 5.42% was observed in 2023 compared to 2021. In 2023 compared to 2022, an increase was observed in January and February and during spring by 1%. In 2022, MCHC showed a decrease in January and February, a decrease of 3.69% during spring and an increase in June, July and September compared with 2021. In 2023, an increase in January, February and July and a percentage increase of 7.82% was observed during spring compared to 2021. A percentage increase of 3.83 and 6.44% was observed in 2023 during spring and autumn respectively, compared to 2021. A significant increase in MCHC was observed in 2023 during January, February, June and July compared to 2021. White blood cells showed decreasing values in April and October during 2022 compared to 2021 and decreasing values in April and June during 2023 compared to 2021. Lymphocytes showed decreasing values in April during 2022 and in June during 2023 compared to 2021. In 2023 increasing values were observed for Neutr in February compared to 2021 and decreasing values compared to 2022. Monocytes showed decreasing values in April, June, July, October and November during 2022 compared to 2021 and decreasing values in July and August and 13.22% decreasing during spring period compared to 2021. Over the 3-year period, Neutr values are positively correlated with seasonal variations in environmental temperatures to which the horses were subjected. Furthermore, environmental temperature was inversely related to seasonal fluctuations of Lymph, RBC, HGB and HCT. Relative humidity was positively associated to the seasonal variations observed for Lymph and negatively correlated with Neutr. The non-significant variation during the different seasons and years indicated that horses were adapted to different climatic conditions without being influenced. For this reason, changes in hematological values are essential in determining the adaptation of animals to the environment (31, 32). Adaptation of the physiological status of animals toward seasonality as observed by the present study depends on the degree of climate change. Therefore, it is necessary to pay attention to the degree of adaptation of the physiological parameters we analyzed even at the diagnostic level, which may lead to an imbalance of the subject’s immune status, states of anemia, electrolyte or protein imbalance as observed in bulls and athletic horses, considering the exercise influence (66, 67). This information underscores the need for season-specific health management strategies in domestic animals (68, 69).

Therefore, it is necessary to provide practical management guidance for the sport horse especially in order to safeguard the state of health and athletic performance by, for example, performing exercise during specific hours of the day, ensure spacious and ventilated shelters to manage heat stress as well as implement sheltered shelters in innovative facilities to cope with a wide variety of external weather conditions considering future climatic alterations due to global warming such as floods, tornadoes, hail or fires. The present study offers a preliminary approach to the impact of climate change on physiological parameters in horses. Therefore, it would be advisable in future studies to extend the monitoring period to highlight a more significant impact of climate change on both hematological parameters indicating the subject’s health status and on more specific blood parameters that could indicate tissue damage, organ damage, and more specific effects due to environmental adaptation.

## 5 Conclusion

Although no significant variation between monitoring years was found for environmental parameters, certain significant variations in hematological parameters were observed over the 3-year period. The climatic changes showed slight increases in environmental temperatures, fortunately not significant for the 3 years analyzed. Similarly, the hematological variations observed from 2021 to 2023, correlated with the environmental parameters, showed significant variations that were nevertheless comforting as they remained within the horse’s physiological range. This preliminary study of 3-year period cannot allow to assess a proper long-term climate change effect, nor to be certain of its impact solely on the haemogram values in the horse, but this study might be helpful for providing information on the hematological profile according to seasonal changes. Therefore, an input could be given for future studies on the effects of climate change on the health of domestic animals as well as in humans and the productive economic impact thereof.

## Data Availability

The raw data supporting the conclusions of this article will be made available by the authors, without undue reservation.
